# Supplementary material for: Magnetic field expulsion in optically driven YBa2Cu3O6.48
Source: Nature. 2024 Jul 10;632(8023):75–80. doi: 10.1038/s41586-024-07635-2 (PMC11291272; doi:10.1038/s41586-024-07635-2)
Supplement: Supplementary file 1 — Supplementary Information [file 41586_2024_7635_MOESM1_ESM.pdf]

nature portfolio

https://doi.org/10.1038/s41586-024-07635-2

Supplementary information

Magnetic field expulsion in optically driven $YBa_2Cu_3O_{6.48}$

In the format provided by the authors and unedited

Magnetic field expulsion in optically driven $YBa_2Cu_3O_{6.48}$

S. Fava[1,*], G. De Vecchi[1,*], G. Jotzu[1,*], M. Buzzi[1,*], T. Gebert[1], Y. Liu[2],

B. Keimer[2], A. Cavalleri[1,3]

[1] Max Planck Institute for the Structure and Dynamics of Matter, 22761 Hamburg, Germany

[2] Max Planck Institute for Solid State Research, 70569 Stuttgart, Germany

[3] Department of Physics, Clarendon Laboratory, University of Oxford, Oxford OX1 3PU, United Kingdom

e-mail: andrea.cavalleri@mpsd.mpg.de, gregor.jotzu@mpsd.mpg.de, michele.buzzi@mpsd.mpg.de

Supplementary Information

S1. Sample Growth and Characterization 2

S2. Experimental Setups and Data Acquisition 2

S3. Data Reduction and Analysis 5

S4. GaP (100) as a Magneto-Optic Detector 8

S5. Additional Temperature Dependence in $YBa_2Cu_3O_{6.48}$ 10

References (Supplementary Information) 12

* These authors contributed equally to this work

S1. Sample Growth and Characterization

The optimally doped $YBa_2Cu_3O_7$ thin films were obtained through a commercial supplier (Ceraco GmbH) and grown on R-cut Al_2O_3 substrates. The films had a thickness of approximately 150 nm, a sharp superconducting transition temperature at around 85 K and critical current density greater than 2 MA/cm^2.

The $YBa_2Cu_3O_{6.48}$ single crystals had typical dimensions of ~2 x 2 x 0.5 mm^3 and were grown in yttrium-stabilized zirconium crucibles. The hole doping of the Cu-O planes was adjusted by controlling the oxygen content of the CuO chain layer by annealing in flowing O_2 and subsequent rapid quenching. A superconducting transition at T_C = 55 K was determined by SQuID DC magnetization measurements, as shown in Fig. S1.

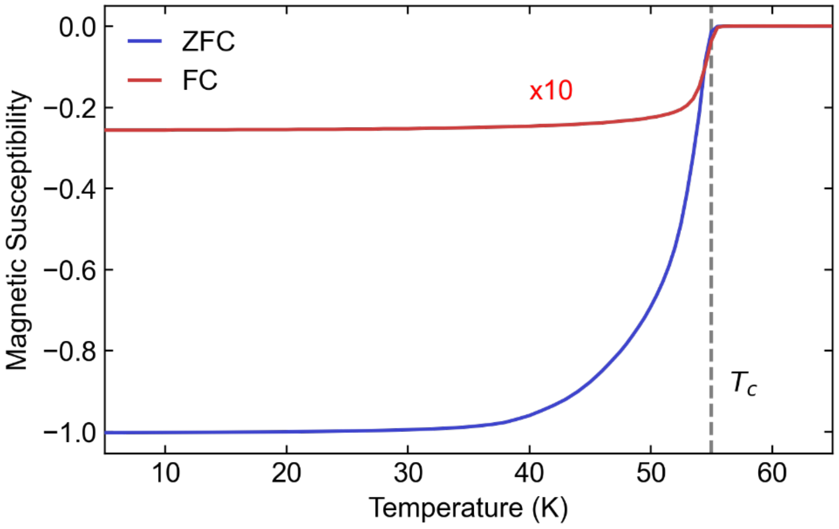

Figure S1. | Temperature dependent DC magnetization measurements (ZFC: zero field cooled, FC: field cooled) highlighting the superconducting transition in $YBa_2Cu_3O_{6.48}$. The measurements were performed in a 1mT applied field perpendicular to the crystal c-axis.

S2. Experimental Setups and Data Acquisition

The equilibrium spatial scans and superconductivity disruption measurements shown in Fig. 1 of the Main Text were performed using the experimental setup sketched in Fig. S2.1. Ultrashort (100 fs) 800 nm laser pulses were produced starting from a commercial Ti:Al_2O_3 oscillator/amplifier chain that produced pulses with energies up to 2 mJ at a repetition rate of 900 Hz. These pulses were split using a beamsplitter into two branches. The lowest intensity branch was used after attenuation for probing the polarization rotation

in the GaP (100) magneto-optic detector. To minimize the noise sources in the measurement, the polarization of the beam was set using a nanoparticle high-extinction ratio linear polarizer.

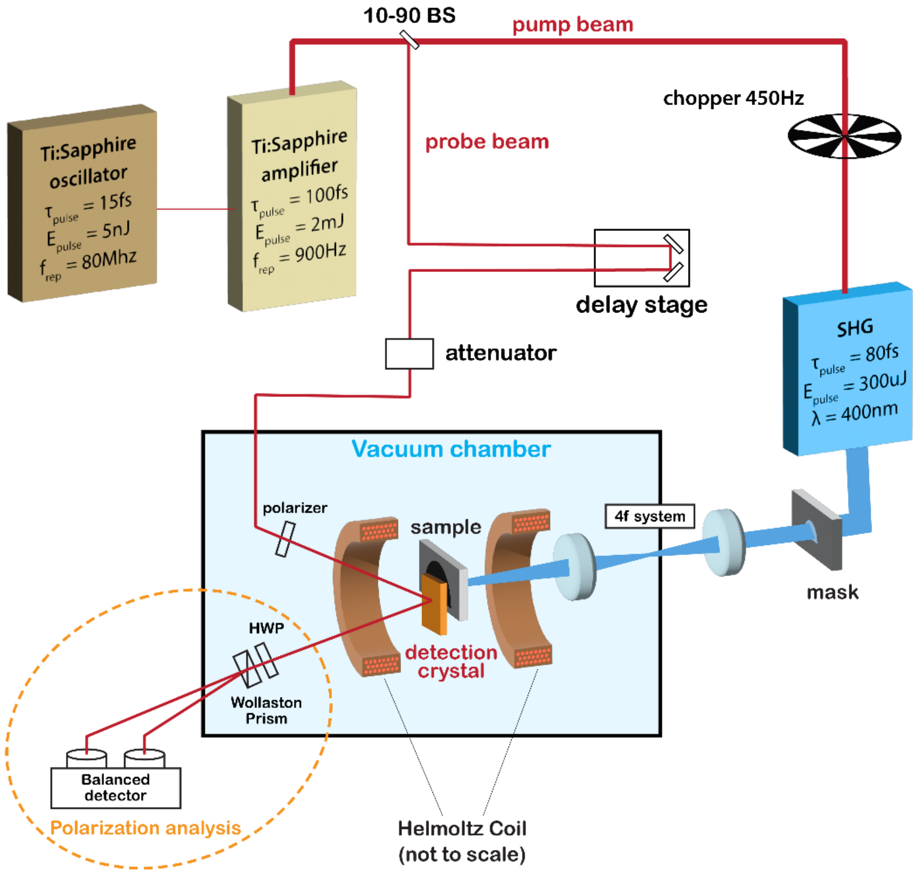

Figure S2.1. | Experimental setup used for the superconductivity disruption measurements shown in Fig. 1 of the Main Text.

As non-normal incidence reflections introduce a phase delay between *s* and *p* polarization, incidence angle fluctuations can give rise to polarization noise. To minimize this, only reflections close to normal incidence were used in the setup and a commercial system using active feedback was used stabilize the laser beam pointing. After traversing and being reflected from the second surface of the Faraday detector, the polarization state of light was analyzed using a half-waveplate, Wollaston prism and balanced photo-diode setup that allowed us to quantify the Faraday effect in the magneto-optic detection crystal. The higher intensity branch was mechanically chopped at a quarter of the repetition rate (225 Hz) and frequency doubled to obtain 400 nm pulses using a β-BaB_2O_4 (BBO) crystal that were used to photo-excite the $YBa_2Cu_3O_7$ thin film samples. A mask, illuminated by these ultraviolet pulses, was imaged onto the back surface of the sample to create a half-gaussian beam with an edge that matched the long edge of the half disc shaped $YBa_2Cu_3O_7$

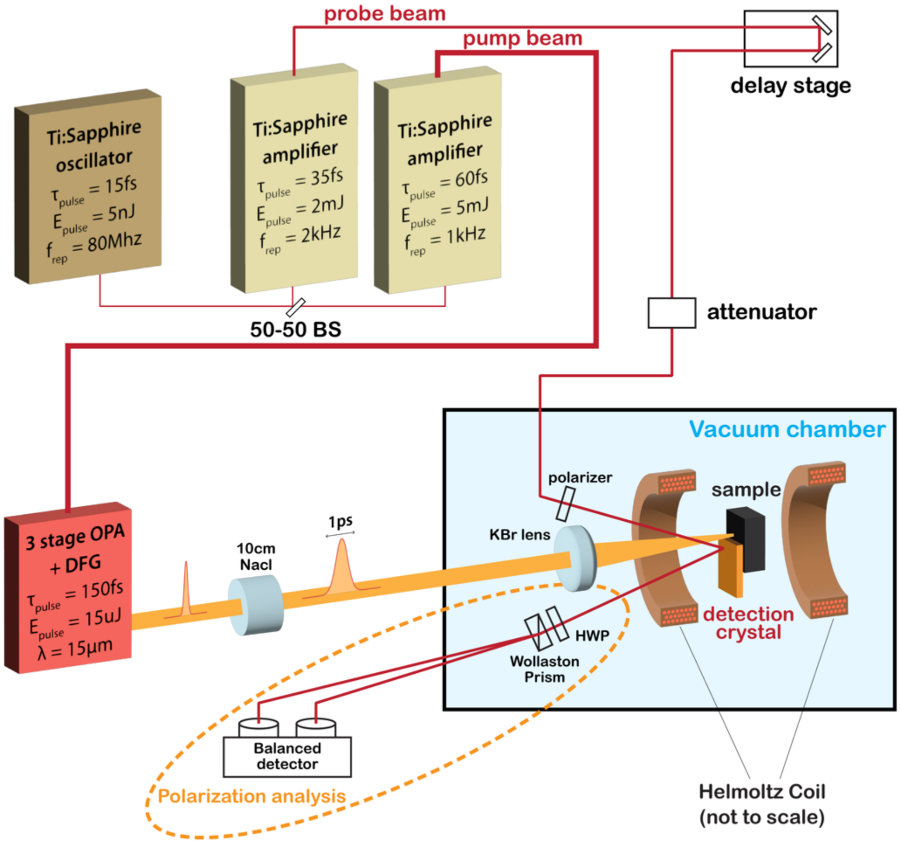

Figure S2.2. | Experimental setup used for the mid-infrared pump, Faraday effect probe measurements shown in Fig. 2, 3 and 4 of the Main Text .

sample. This, together with $YBa_2Cu_3O_7$ being fully opaque to 400 nm radiation ensured that the GaP detector was not exposed to the pump pulses.

The $YBa_2Cu_3O_7$ thin film samples were embedded in the detector assembly (Extended Data Fig. 2) and mounted on the cold finger of a liquid helium cryostat to allow for temperature control. The cryostat was directly placed in a high vacuum chamber that contained also the part of the optical setup dedicated to polarization analysis to avoid spurious contributions arising from vacuum windows. A pair of coils in a Helmholtz configuration generated a magnetic field at the sample position whose polarity could be reversed at a frequency of 450 Hz. The highest achievable magnetic field was limited by heat dissipation and was ~3 mT. The sample position was controlled using computer controlled linear translation stages that made it possible to reproducibly move the cryostat and the sample inside the vacuum chamber with ~10 μm repeatability.

The measurements shown in Fig. 2, 3, and 4 were carried out with a different experimental setup sketched in Figure S2.2. Here, 800 nm pulses were generated using a pair of

commercial Ti:Al_2O_3 amplifiers seeded by the same oscillator to achieve femtosecond synchronization. One amplifier produced 35 fs long, ~2 mJ pulses at 2 kHz repetition rate and was used for the probe beam. The second amplifier produced ~60 fs long, ~5 mJ pulses at 1kHz repetition rate and was used to pump a home built three stage OPA that generated ~2mJ total energy signal and idler pulses. These pulses were mixed in a 0.4mm thick GaSe crystal to obtain ~150 fs long, ~20 μJ energy pulses centered at ~20 THz, close to resonance with the B_{1u} apical oxygen phonon modes of $YBa_2Cu_3O_{6.48}$. These pulses were then chirped using a 10mm NaCl rod to a duration of ~1 ps, in order to match the optimum pulse length for inducing superconducting-like optical properties in $YBa_2Cu_3O_{6.48}$.[1] While the sample stages and cryostat were similar between the two setups, in this case the polarization analysis setup is fully "in-line", i.e. the beam travels directly from the polarizer to the Wollaston analyzer without being reflected by additional mirrors other than the detector. This contributed to further reduce spurious sources of polarization noise. A magnetic field was applied at the sample position using a pair of Helmholtz coils whose polarity was switched at ~10 Hz frequency and could reach a maximum amplitude of 12.5 mT.

In both experimental setups the polarity of the magnetic field is cycled periodically at a sub-harmonic of the pump and probe repetition rates. To obtain differential pump-probe measurements the electrical pulses from the balanced photodetector were digitized using a commercial 8 channel 40MS/s data acquisition card, triggered at the lowest frequency used in the experiment. These signals, acquired in the time-domain, were then integrated, after applying boxcar functions, yielding the amplitude of the signal from the sum and difference channels of the balanced photodetector for each probe laser pulse. Since the acquisition of a full pulse sequence required the acquisition of many pump-probe cycles, the sample clock signal of the data acquisition card is derived using direct digital synthesis from the oscillator repetition rate. In this way drifts in the cavity length and repetition rates of the system do not affect the relative timing of the boxcar functions with respect to the arrival time of the electrical pulse.

S3. Data Reduction and Analysis

As mentioned in the previous section for all the experiments the polarity of the magnetic field was cycled periodically and measurements with pump and without pump were acquired to yield double differential pump probe measurements and isolate contribution to

the polarization rotation that were induced by the applied magnetic field. This allowed us to filter out all contribution to the polarization rotation that would not invert with the externally applied magnetic field. In other words, because the pump-induced magnetic field changes measured with applied field $-B_{\text{ext}}$ were subtracted from those acquired with applied field $+B_{\text{ext}}$, the signal is not sensitive to magnetic fields carried by the MIR pulse itself or due to transient generation of fast electrons in the sample. In the following we discuss this approach in detail and the impact it has on the measured quantities.

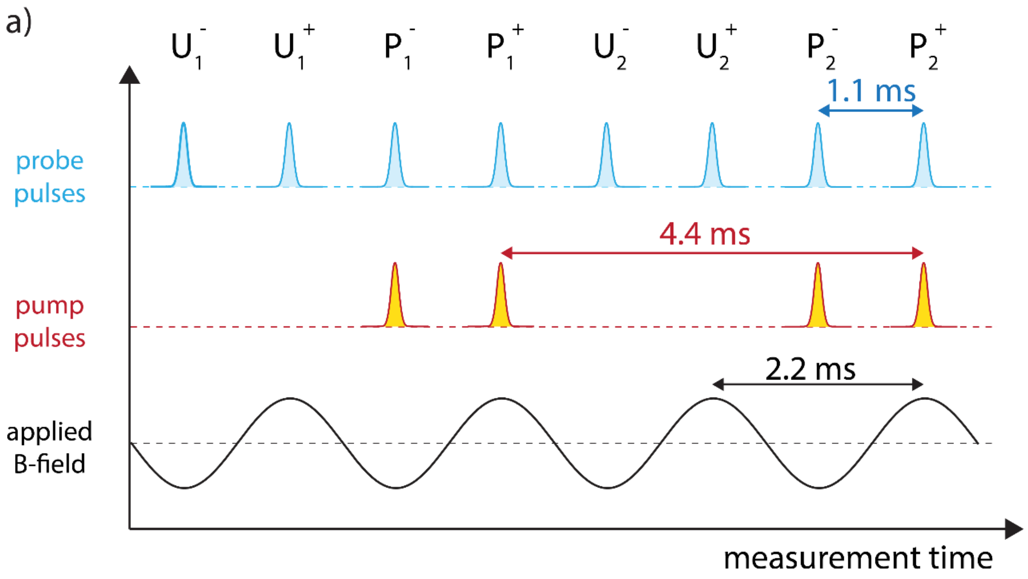

Figure S3.1. | Timing diagram of the acquisition scheme used for the superconductivity disruption measurements shown in Fig. 1 of the Main Text.

For the measurements shown in Fig. 1 of the Main Text the magnetic field polarity was cycled following a sinewave at 450 Hz frequency and the pump was mechanically chopped at 225 Hz. A timing diagram of the acquisition scheme is shown in Fig. S3.1. The amplitude of the signal of the balanced photodetector difference channel is normalized by that of the sum channel in a pulse-by-pulse manner. For convenience we label these signals as $U_i^{\pm}$ to indicate those acquired with the pump off for positive and negative polarities of the applied magnetic field and $P_i^{\pm}$ to indicate the same signals acquired with the pump on. The subscript *i* runs over the n repetitions in the acquisition. The following quantities are calculated as follows:

$$\Delta\vartheta_{\text{pump-off, i}} = U_i^+ - U_i^- \qquad \Delta\vartheta_{\text{pump-on, i}} = P_i^+ - P_i^- \qquad \Delta\vartheta_{\text{pp}} = \Delta\vartheta_{\text{pump-on}} - \Delta\vartheta_{\text{pump-off}}$$

where $\Delta\vartheta_{\text{pump-off}}$ and $\Delta\vartheta_{\text{pump-on}}$ (averaged over n repetitions) are the magnetic field induced polarization rotations measured with the pump off and on respectively and $\Delta\vartheta_{\text{pp}}$ is the magnetic field induced change in polarization rotation due to the pump. These quantities yielded the amplitude of the magnetic field and its pump-induces changes, after calibration of the Faraday effect in the GaP (100) detector (see Supplementary Information S4). To cancel out residual drifts, the phase of the magnetic field, as well as that of the pump laser, with respect to the probe laser, are periodically alternated between 0 and π.

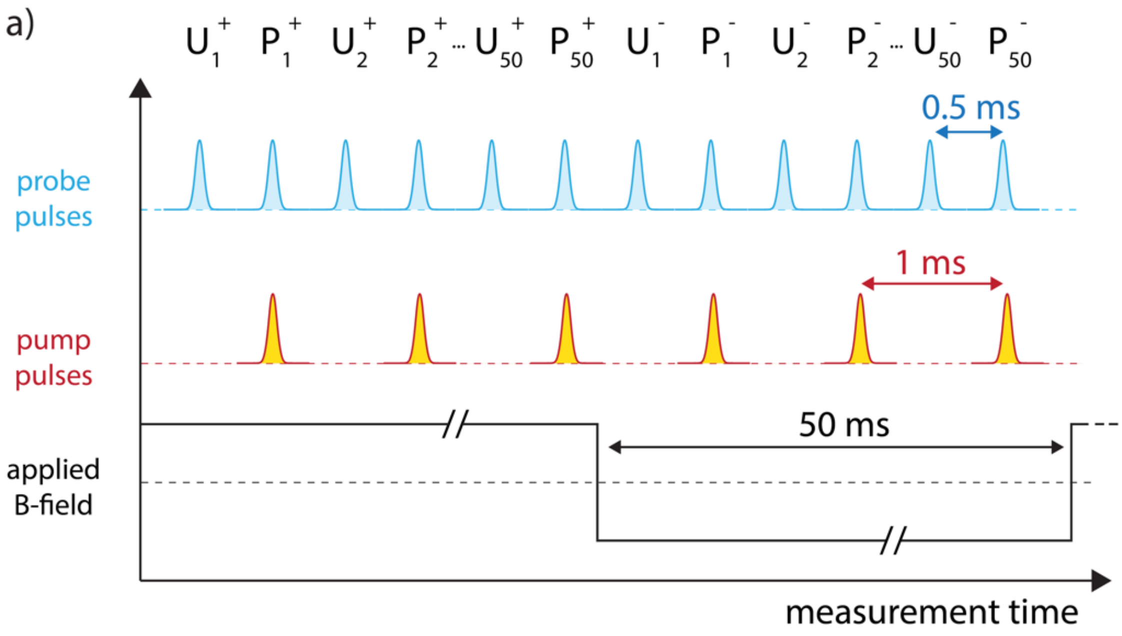

Figure S3.2. |Timing diagram of the acquisition scheme used for the mid-infrared pump, Faraday effect probe measurements shown in Fig. 2, 3, and 4 of the Main Text.

The measurements reported in Fig. 2, 3, and 4 of the Main Text were acquired using a slightly different scheme to ensure that the sample was excited in a constant magnetic field. Here the probe repetition rate was 2 kHz and the pump struck the sample every second probe pulse (i.e. at 1 kHz) while the magnetic field polarity was modulated following a square wave at a lower frequency of around ~10 Hz. This ensured that the sample was photoexcited in a constant magnetic field. The same quantities as described above were calculated yielding double-differential pump probe measurements that distilled only the contributions to the polarization rotation arising from pump-induced changes in the magnetic properties of the sample.

S4. GaP (100) as a Magneto-Optic Detector

The ultrafast optical magnetometry technique we introduced in the Main Text relies on the Faraday effect which directly relates the magnetic field applied to a material to the polarization rotation of a linearly polarized beam traversing the medium. This relation is normally reported as:

$$\theta = VBL$$

where θ represents the rotation of the polarization of the input beam, B is the magnitude of the magnetic field along the light propagation direction inside the medium and L is the thickness of the medium. The proportionality constant V is known as the Verdet constant, which is a material dependent constant depending also on other parameters such as the wavelength of the incoming polarized light.

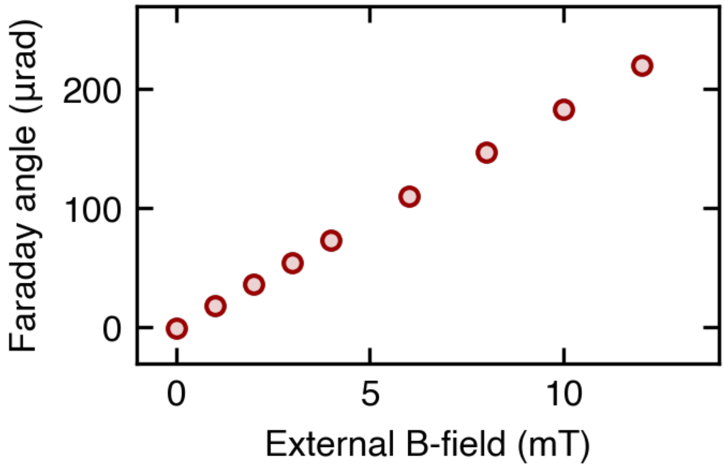

Figure S4.1. |Sensitivity calibration measurement of the 70 µm thick GaP (100) detectors used for the measurements presented in this work. The measurements were performed at a temperature T = 100K as a function of the applied magnetic field.

In the past, the Faraday effect in ferromagnetic crystals and thin films has been used to image the magnetic properties of superconductors at equilibrium.[2,3] While these types of detectors (such as $Bi:Y_3Fe_5O_{12}$, EuS, and EuSe) offer very high sensitivity ($V\sim 10^5$ rad·T^{-1}·m^{-1}) they have limited time resolution, down to 100 ps at best, due to the presence of low lying magnetic excitations (e.g. ferromagnetic resonance) at sub-THz frequencies. Diamagnetic II-VI and III-V semiconductors such as ZnSe, ZnTe and GaP have a magneto-optic response featuring Verdet constants that are two to three orders of magnitude smaller than those observed in ferromagnetic materials. Although less sensitive, these

materials have the advantage of not being magnetically ordered and offer significantly better time resolution[4,5]. Furthermore, their Verdet constant is mostly temperature independent ensuring a flat detector response in a broad temperature range.
The measurements shown throughout the manuscript were performed using GaP detectors prepared as detailed in Extended Data Fig. 2. The sensitivity of these detectors was calibrated using the same polarization analysis setup used for the measurements. This was done recording the field-induced polarization rotation at different known applied magnetic fields. To do so, the in-situ Helmholtz coil pair was independently calibrated using a Lakeshore 425 gaussmeter. The results of this calibration measurement, performed at 100 K are reported in Fig. S4.1 and accounting for the detector thickness yielded a Verdet constant of ~120 $\mathrm{rad \cdot T^{-1} \cdot m^{-1}}$, in agreement with reported literature values[6].
Since GaP is an optically isotropic material, the Faraday effect is also expected to be isotropic and its strength only depends on the orientation of the light propagation direction with respect to the magnetic field inside the crystal. Hence, the measured magnetic field-

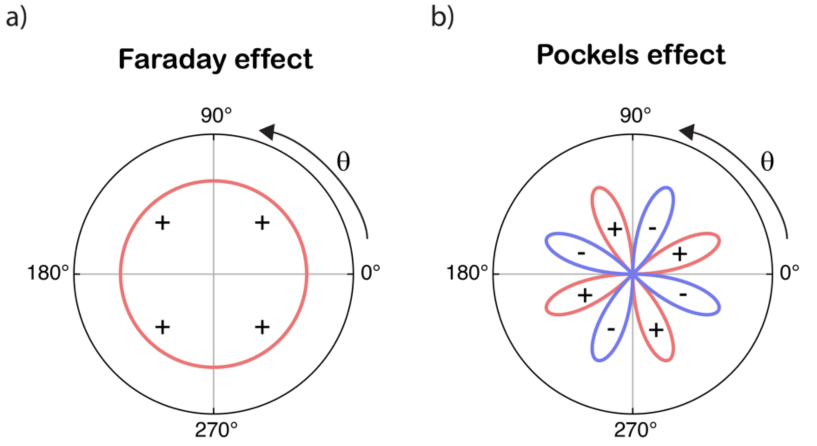

Figure S4.2. | **(a)** Input polarization dependence of the measured signal expected for the Faraday effect in a GaP (100) crystal under the presence of a magnetic field parallel to the [100] direction. **(b)** Same as in (a) but due to the electro-optic effect, assuming a finite incidence angle and an electric field applied in the (100) plane.

induced polarization rotation is expected to be independent on the input polarization angle with respect to the crystal axes as shown in Fig. S4.2a.
In GaP, inversion symmetry is broken and besides being magneto-optically active it also features an electro-optic (Pockels) effect, that is an electric field induced birefringence. Since ultrafast magnetic field pulses are propagating electromagnetic waves, a time dependent electric field at THz frequencies is also present and care must be taken to distinguish the two effects. In the geometry of our experiment, with an applied magnetic field

perpendicular to the plane of the detector, we expect electric fields associated to ultrafast changes in the local magnetic field to be polarized in the plane of the detector. Based on the symmetry of the electro-optic tensor, electric fields polarized in the plane of a (100) oriented GaP crystal will not cause any birefringence for probe beams propagating along the [100] crystal axis.

Small contributions due to misalignment of the crystal orientation, finite incidence angles, or electric fields polarized out of the (100) plane will give rise to a signal at the balanced photo-detector that depends on the angle of the probe beam input polarization, due to symmetry. These contributions can be calculated extracting the field induced birefringence from the eigenvalues and eigenvector of the dielectric impermeability tensor and calculating the expected signal using Jones calculus[7]. For example, in Fig. S4.2b we report the polarization dependence of the photo-detector signal that is expected to be observed with an electric field in the (100) plane and a finite incidence angle. The signal should exhibit an eight-fold dependence on the input polarization. This is in contrast with the polarization dependence reported in the inset of Fig. 2b and corroborates that the measured signal originated from a Faraday effect.

S5. Additional Temperature Dependence in $YBa_2Cu_3O_{6.48}$

Additional data were collected in the same geometry as Fig. 2 at temperatures lower than 100 K and also at T=45 K< T_c. The extended temperature dependence is shown in Fig S5.1. Note that these data are reported normalized by the peak 100 K value since they were acquired with a different detector (ZnTe (100)) compared to those reported in the Main Text.

The observed magnetic field expulsion appears to saturate below 100K. At T=45 K< T_c, where $YBa_2Cu_3O_{6.48}$ is already diamagnetic, we observe an enhanced expulsion. This result is broadly compatible with previous results from MIR pump, THz probe experiments revealing an enhancement of the superconducting properties also below T_c.[8]

However, the enhancement observed at 45 K should be interpreted with caution. As shown in Fig. 5.2, when one excites the normal state, one induces a superconducting-like thin layer above a non-superconducting (and weakly paramagnetic) bulk. This makes the measurements of Fig. 2 very "clean", as the magnetic response of the thin photo-excited layer dominates the overall evolution of the field outside the sample. This is not the case

when exciting below T_c. There, a diamagnetic equilibrium superconductor is present beneath the photo-excited layer (see Fig. S5.2) and is likely to affect the magnetic field dynamics after photo-excitation making it difficult to quantitatively compare data acquired below and above T_c.

We envisage that future experiments on thin *lamellae* of c-axis cut $YBa_2Cu_3O_{6.48}$, having a thickness comparable to the penetration depth of the pump will create an experimental setting in which a reliable comparison of measurements below and above T_c will be possible.

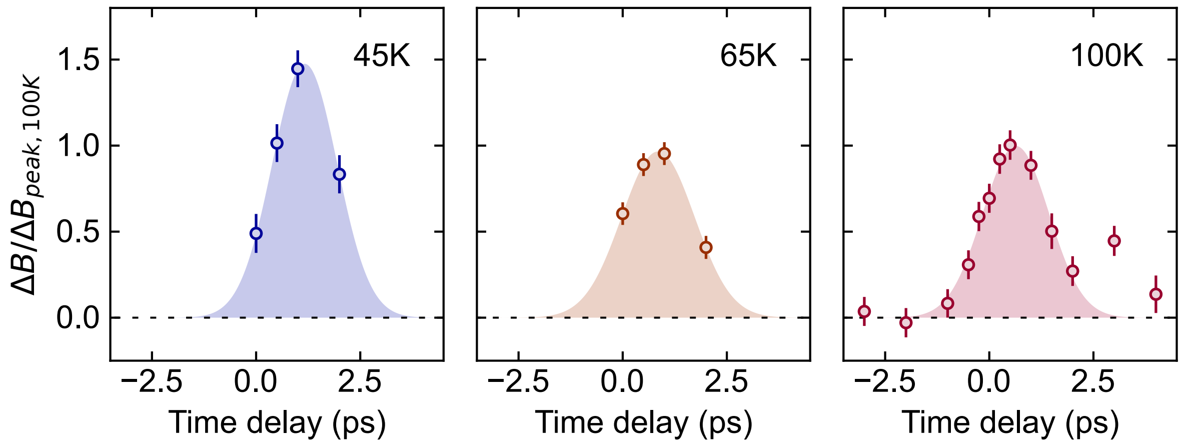

Figure S5.1. | Pump-induced changes in the measured magnetic field (ΔB) as function of pump-probe delay measured at three different temperatures of 45 K (blue), 65 K (orange), and 100 K (red). The values are normalized by the 100 K peak value since these measurements were performed with a different detector (ZnTe (100)) compared to those reported in the main text.

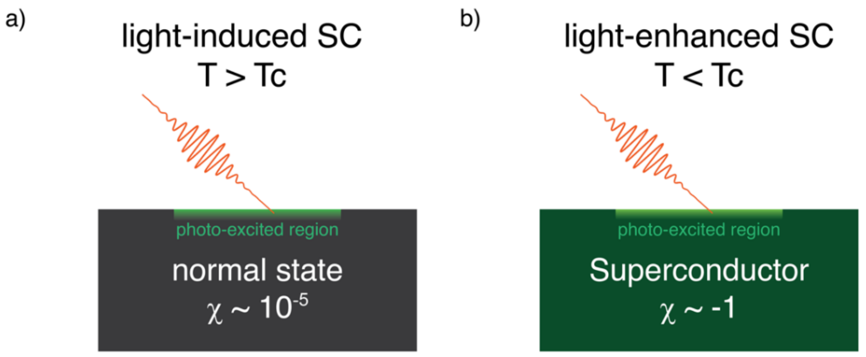

Figure S5.2. | Different boundary conditions for the experiments performed above and below Tc. **(a)** Above Tc, the photoexcited region (light green) is surrounded by YBCO in its normal state which shows a very weak magnetic response. **(b)** Below Tc the photoexcited region is surrounded by YBCO in its superconducting state featuring a strong diamagnetic response.

References (Supplementary Information)

1. Ribak, A. *et al.* Two-fluid dynamics in driven $YBa_2Cu_3O_{6.48}$. *Phys. Rev. B* **107**, 104508 (2023).
2. Goa, P. E., Hauglin, H., Olsen, Å. A. F., Baziljevich, M. & Johansen, T. H. Magneto-optical imaging setup for single vortex observation. *Rev. Sci. Instrum.* **74**, 141–146 (2003).
3. Koblischka, M. R. & Wijngaarden, R. J. Magneto-optical investigations of superconductors. *Supercond. Sci. Technol.* **8**, 199 (1995).
4. Riordan, J. A., Lu, Z. G. & Zhang, X.-C. Free-Space Ultrafast Magneto-Optic Sampling. in *Conference on Lasers and Electro-Optics (1997), paper CPD10* CPD10 (Optica Publishing Group, 1997).
5. Riordan, J. A. & Zhang, X.-C. Sampling of free-space magnetic pulses. *Opt. Quantum Electron.* **32**, 489–502 (2000).
6. Parsons, D. F. & Coleman, P. D. Far Infrared Optical Constants of Gallium Phosphide. *Appl. Opt.* **10**, 1683_1-1685 (1971).
7. Allen, S. Electro-optic Materials and Applications. in *Electronic Materials: From Silicon to Organics* (eds. Miller, L. S. & Mullin, J. B.) 301–313 (Springer US, Boston, MA, 1991).
8. Liu, B. *et al.* Pump Frequency Resonances for Light-Induced Incipient Superconductivity in $YBa_2Cu_3O_{6.5}$. *Phys. Rev. X* **10**, 011053 (2020).
